# The Effect of Online Violent Video Games on Levels of Aggression

**DOI:** 10.1371/journal.pone.0111790

**Published:** 2014-11-12

**Authors:** Jack Hollingdale, Tobias Greitemeyer

**Affiliations:** 1 School of Psychology, University of Sussex, Brighton, United Kingdom; 2 Institute of Psychology, University of Innsbruck, Innsbruck, Austria; Brock University, Canada

## Abstract

**Background:**

In recent years the video game industry has surpassed both the music and video industries in sales. Currently violent video games are among the most popular video games played by consumers, most specifically First-Person Shooters (FPS). Technological advancements in game play experience including the ability to play online has accounted for this increase in popularity. Previous research, utilising the General Aggression Model (GAM), has identified that violent video games increase levels of aggression. Little is known, however, as to the effect of playing a violent video game online.

**Methods/Principal Findings:**

Participants (*N* = 101) were randomly assigned to one of four experimental conditions; neutral video game—offline, neutral video game—online, violent video game—offline and violent video game—online. Following this they completed questionnaires to assess their attitudes towards the game and engaged in a chilli sauce paradigm to measure behavioural aggression. The results identified that participants who played a violent video game exhibited more aggression than those who played a neutral video game. Furthermore, this main effect was not particularly pronounced when the game was played online.

**Conclusions/Significance:**

These findings suggest that both playing violent video games online and offline compared to playing neutral video games increases aggression.

## Introduction

The video game industry is now the largest entertainment industry in the UK. 2011 industry figures have identified that game sales, including platform and digital, have exceeded both music and video sales [1]. Violent video games have previously been identified to be the most popular video games played by consumers [2]. Research into the effect of violent video games on levels of aggression has led to concerns that they may pose a public health risk [3]. Indeed, cross-sectional studies have found positive correlations between violent video game play and real-life aggression [4]–[6]. Longitudinal studies showed that habitual violent video game play predicts later aggression even after controlling for initial levels of aggressiveness [7]–[9]. Finally, experimental studies have revealed that playing violent video games is a causal risk factor for increased aggression [10]–[12]. It should be noted, however, that there is other research showing no evidence that engagement with violent video games leads to increases in aggression or reductions in prosocial behaviour [13]–[16], warranting the need for further research in this area. On balance however, evidence from meta-analyses confirm that exposure to violent video games increases aggressive cognitions, aggressive affect and aggressive behaviour, and decreases empathy and prosocial behaviour [17], [18].

Much of the research that has provided evidence to indicate the negative effects of violent video games has utilised the General Aggression Model (GAM) [19]. A widely accepted model for understanding media effects, the GAM posits that cognition, affect and arousal mediate an individual's perception of a situation. Thus, in the short term a violent video game may temporarily increase aggression through the activation of one or more of these domains. In the long term aggressive scripts can develop and become more readily available [4]. Therefore the GAM can explain how properties of a video game can affect players' thoughts, feelings, physiological arousal and subsequent behaviour. Technological developments have afforded such games, and subsequent gaming experience, to expand beyond the realms of the console, and computer programmed opponents (offline gaming), and now allow players to engage in video game play with multiple players from all over the world via the internet (online gaming). Schubert, Regenbrecht and Friedmann [20] found that players who interact with other human players experience a heightened sense of being part of the action. Significant differences in physiological arousal and evaluations of game experience, including presence and likability, have also been found when video game opponents are controlled by other humans [21]. In regards to the negative effects, increases in aggressive thoughts and hostile expectations have been found when playing human opponents in a violent video game [22], [23]. Further to this, Wei [24] found, from a survey of 312 Chinese adolescents, that those who played violent video games online against human opponents expressed a greater tolerance of violence, a lower empathetic attitude and more aggressive behaviour than those who played against computer opponents. Based on previous studies, engagement with/against human opponents may strengthen gaming experiences and therefore, in accordance with the GAM, heighten their effects on players' thoughts, feelings and behaviour.

As noted above, violent content within violent video games has also been identified to increase levels of aggression. Within specific violent video games, progression through gaming levels achieved by engaging in violence poses an additional risk of increasing levels of aggression. Carnagey and Anderson [25] found that rewarding violence increased in-game violence and that rewards for killing other racing drivers and pedestrians, in the race-car video game *Carmageddon 2*, increased levels of hostile emotion, aggressive thinking and aggressive behaviour. Sherry [26] identified that video games that portray human violence were associated with increases in levels of aggression, potentially due to higher rates of action, and subsequent heightened nonspecific arousal. More specifically, increases in experience of perceived difficulty, enjoyment and action have yielded significant game effects on aggressive thoughts [27]. These findings lend support to the processes involved in the GAM.

One of the most popular violent gaming formats to date is the First Person Shooter (FPS), in which the gamer experiences the action through the eyes of the main protagonist, centred on a projectile weapon. Reports indicate that a specific franchise, utilising the FPS format, *Call of Duty*, a military war game, has broken all previous sales records. *Call of Duty: Modern Warfare 2*, made $550 m (£350 m) in the first five days of sale. This was surpassed by *Call of Duty: Black Ops* and *Call of Duty: Modern Warfare 3*, which made $650 m (£412 m) and $775 m (£490 m) in sales respectively [28]. FPSs have been found to significantly increase hostility and aggression from base line levels [29]. Based on anecdotal evidence much of the success of this franchise has been attributed to features of online game play.

Despite the popularity of the genre, to date, there is a lack of research that has attempted to investigate the effect of playing violent video games, specifically FPSs, online on levels of aggression.

### Overview of the present research

In the present research, we examined whether playing a FPS online would exacerbate the negative effects of violent video game play on aggression. Further to this we examined the effect of particular game experiences including perceived difficulty, enjoyment and action, previously identified to be associated with increases in aggressive thoughts [27], on levels of behavioural aggression. To this end, participants played either a violent video game online or offline, or a neutral video game online or offline. Afterwards, aggressive behaviour was assessed. It was expected that playing a violent video game would increase aggression. It was also expected that participants who had played the violent video game online would show the highest levels of aggression (relative to the remaining three experimental conditions) due to the previously identified experiences specific to online game play. Finally, we examined whether these proposed effects would hold when controlling for perceived difficulty, enjoyment and action.

Ethical approval was given by the University of Sussex's School of Life Sciences Research Governance Committee (Ethical Approval Reference: RBJH0510). All relevant data are within the paper and its Supporting Information files.

## Method

Within this paper the authors report how we determined our sample size, all data exclusions (if any), all manipulations, and all measures in the study. One hundred and one students (64 men and 37 women; ages range from 18 to 44: *M* = 21.38, *SD* = 4.00) from a UK University participated in the study in exchange for course credits or payment. After being welcomed by the examiner all participants were asked to complete a consent form. Participants were randomly assigned to one of four experimental conditions; 26 participants in a neutral video game offline, 26 participants in a neutral video game online, 23 participants in a violent video game offline and 26 participants in a violent video game online. Participants were advised that they would be undertaking two unrelated marketing surveys that had been combined for the economy of time. The first would ask for their views about a popular video game and the second would involve a marketing survey for a new recipe of hot chilli sauce.

The first task involved playing a video game for thirty minutes [29] either offline or online. In the offline condition participants were allowed to play against computer characters, subject to the video game's narrative. In the online condition participants played against human opponents via the internet, utilising randomly computer selected pre-existing levels, thus reducing the time spent navigating menus. In the online conditions, when appropriate, participants were requested to wait patiently whilst the server selected and loaded following levels. There was no opportunity for players to communicate with other human players via the internet in the online condition. The audio was turned off in all conditions to prevent participants being exposed to other players' attitudes or opinions in the online condition and to promote consistency. The gaming approach and engagement of online opponents was not recorded. All participants were initially introduced to a Playstation 3 computer console. The type of video game (violent and neutral) was identified using their Pan European Game Information (PEGI) ratings. Participants in the neutral video game condition were introduced to *LittleBigPlanet 2*, certificate 7, a game that would normally be rated suitable for all age groups but contains scenes that may be considered frightening for young children [30]. *LittleBigPlanet 2* allows players to create, explore, solve puzzles, and interact with fantasy environments which they can enjoy or share online with other gamers. All participants in the neutral condition played the initial training level and were then allocated to either the offline condition, subject to the game's narrative, or online condition, able to engage freely with the game's online content, for the remainder of the experiment. Participants in the violent video game condition were introduced to *Call of Duty: Modern Warfare*, certificate 18. *Call of Duty: Modern Warfare* is a FPS that sets gamers as soldiers tasked to kill the enemy in various environments. Games with a certificate 18 depict extreme violence including multiple, motiveless killing and violence towards defenceless people that may make the viewer experience a sense of revulsion [30]. All participants were asked to play the initial level, that introduces players to the gaming controls, and then were set up to play offline levels, following the narrative of the game, or online levels, during which the player played against other human operated opponents in free-for-all mode (Deathmatch). Having played for the allotted time participants were then asked to complete a number of questions about the game they had just played. This survey investigated their attitudes towards the games, including how violent they perceived the content and the graphics to be. Among some filler items, participants indicated how difficult they perceived the game to be (using two items, α = .72), to what extent they enjoyed the game (using two items, α = .79), and how fast the action of the game was (using one item). All items were assessed on a Likert scale from 1 to 7.

Following this, some affective measures were employed. There were no significant effects on these measures so this is not considered further. Finally, participants completed a marketing survey investigating a new hot chilli sauce recipe. Participants were informed that they were not required to taste the hot chilli sauce but to prepare an amount of chilli sauce for a taste tester. During the instructions they were made aware that the taste tester ‘couldn't stand hot chilli sauce’ but was taking part due to good payment. They were presented with a hot chilli sauce, depicting three out of three chillies for hotness, a spoon and a plastic receptacle. The amount of chilli sauce was weighed in grams after the participant had left the experiment. The chilli sauce paradigm has been successfully used in previous studies to measure behavioural aggression in the laboratory environment [31]. All participants completed all parts of the experiment with none admitting to knowing the true purpose of the study, therefore all data was included within the study. At the conclusion of the experiment all participants were offered a comprehensive debrief form which included information as to the true purpose of the experiment.

## Results

The manipulation check identified that participants in the violent video game condition reported that the violent video game *Call of Duty: Modern Warfare 2* (*M* = 4.08, *SD* = 1.29) depicted a more violent content and more violent graphics compared to the neutral video game *LittleBigPlanet 2* (*M* = 1.41, *SD* = 0.89), *F*(1, 97) = 146.97, *p*<.001, η_p_^2^ = .60.

A 2 (type of video game: violent vs. neutral) x 2 (setting: online vs. offline) analysis of variance (ANOVA) on the amount of chili sauce (aggression measure) revealed a significant main effect of type of video game, *F*(1, 97) = 8.63, *p* = .004, η_p_^2^ = .08. Participants who had played the violent video game were more aggressive (*M* = 16.12, *SD* = 15.30) than participants who had played the neutral video game (*M* = 9.06, *SD* = 7.65). The main effect of setting, *F*(1, 97) = 0.35, *p* = .558, η_p_^2^ = .00, and the interaction were not significant, *F*(1, 97) = 1.44, *p* = .234, η_p_^2^ = .02.

**Figure 1:**
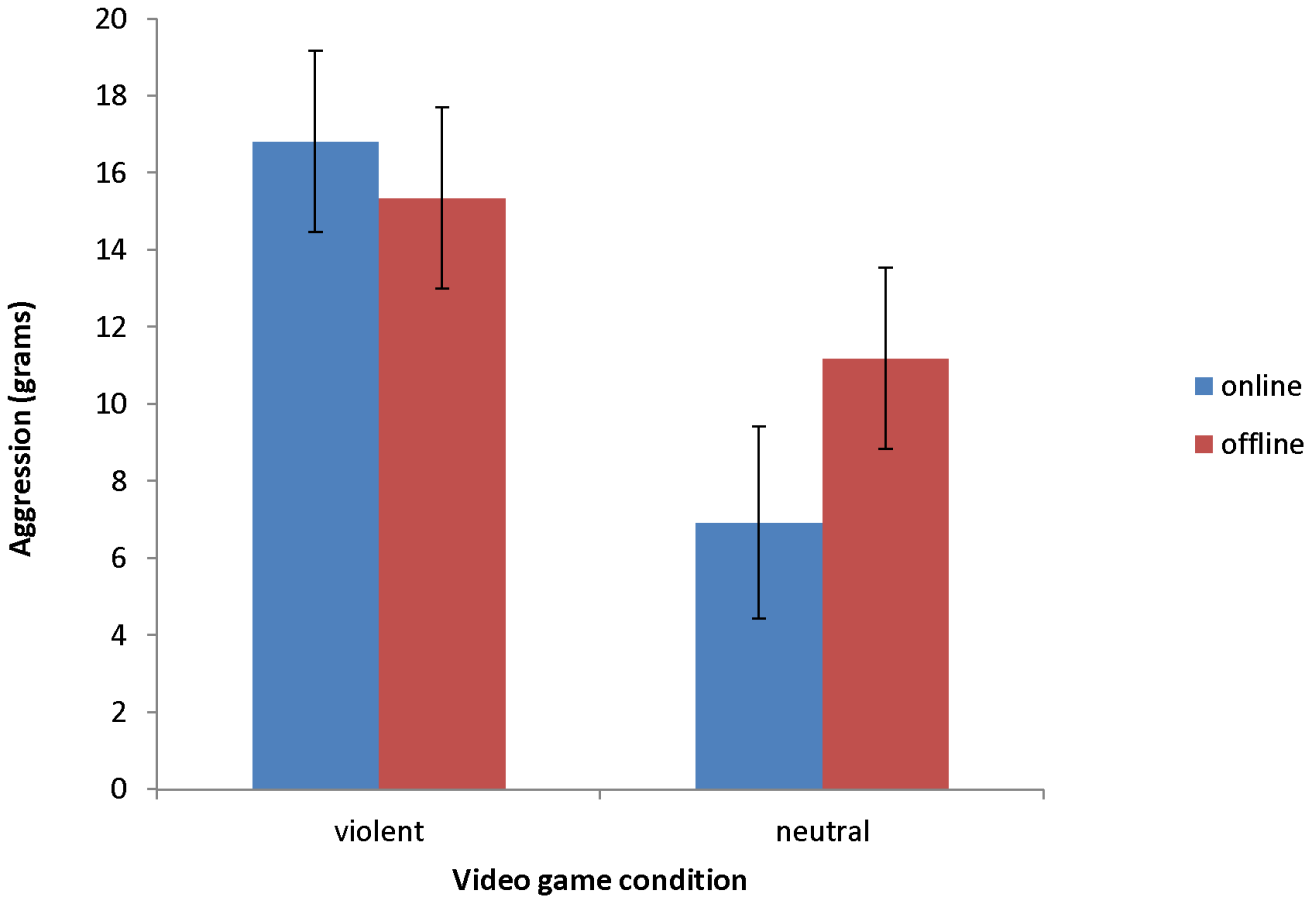
Mean grams of chilli sauce by experimental condition.

To test our specific prediction that aggressive behaviour, grams of chilli sauce dispensed by participants, would be particularly pronounced after playing a violent video game online, planned contrasts were performed, which are particularly adequate to answer such specific research questions [32], [33]. In fact, participants who had played the violent video game online were more aggressive (*M* = 16.81, *SD* = 16.57; contrast weight: 3) compared to participants who had played the violent video game offline (*M* = 15.35, *SD* = 14.04; contrast weight: −1), participants who had played the neutral video game online (*M* = 6.92, *SD* = 7.62; contrast weight: −1), and participants who had played the neutral video game offline (*M* = 11.19, *SD* = 7.20; contrast weight: −1), *t*(97) = 2.07, *p* = .041 (Figure 1). Note, however, that the orthogonal contrast comparing the violent video game offline condition (contrast weight: 2) with the neutral video game online (contrast weight: −1) and the neutral video game offline (contrast weight: −1) condition was also significant, *t*(97) = 2.09, *p* = .039. Finally, the orthogonal contrast comparing the neutral video game online (contrast weight: 1) with the neutral video game offline (contrast weight: −1) condition was not significant, *t*(97) = 1.28, *p* = .202. This pattern of data suggests that both playing violent video games online and offline compared to playing neutral video games increases aggression.

The violent video game (*M* = 4.11, *SD* = 1.48) was perceived as being more difficult than the neutral game (*M* = 2.71, *SD* = 1.18), *F*(1, 97) = 27.11, *p*<.001, η_p_^2^ = .22. Participants also enjoyed the violent video game more (*M* = 4.81, *SD* = 1.46) than the neutral game (*M* = 3.76, *SD* = 1.38), *F*(1, 97) = 13.34, *p*<.001, η_p_^2^ = .12. The violent video game (*M* = 5.00, *SD* = 1.47) was also perceived as having faster action than the neutral video game (*M* = 2.94, *SD* = 1.56), *F*(1, 97) = 46.06, *p*<.001, η_p_^2^ = .32. Note, however, that in a multiple regression the effect of type of video game (violent vs. neutral) was still significant when controlling for these video game ratings, β = .27, *t*(96) = 2.15, *p* = .034. Moreover, none of the video game ratings received a significant regression weight, all βs<.15, all *ts*<1.29, all *ps*>.202.

## Discussion

The present study examined the effect of playing a violent video game online and the impact of game experience including perceptions of difficulty, enjoyment and action on levels of behavioural aggression. Supporting previous research, this study found that playing a violent video game in comparison to a neutral video game significantly increased levels of aggression [3]–[6]. However, this main effect was not particularly pronounced when the game was played online. That is, both playing the violent video game online and offline relative to playing a neutral video game increased levels of aggression.

It is important to note that the violent and the neutral video game differed in terms of perceived difficulty, enjoyment and action, with the violent game perceived as being more difficult, more enjoyable, and being faster. However, when controlling for these video game properties, there was still a significant influence of type of video game on aggression. To put it differently, the effect that playing the violent relative to the neutral video game increases aggression is *not* due to differences in perceived difficulty, enjoyment and action. It should be noted, however, that controlling for potential confounders within video game research should be viewed with caution [34].

It should be acknowledged that the violent and the neutral video game chosen for this study may differ in properties other than difficulty, pace of action, and enjoyment. For example, the first-person shooter game, even when played offline (alone), contains a great deal of competitive content (competing in shooting battles for survival against other computer-generated characters), whereas the neutral video game contains little to no competitive content. Importantly, previous research has demonstrated an effect of competitive video game content (i.e., competing against other computer-generated characters in a game) on aggressive behavior in the short-term [35] and long-term [36]. Unfortunately, we did not control for competitive content so it may well be that our finding that violent video games increase aggression can be (in part) accounted for by differences in how competitive the game is perceived to be. This is certainly an important endeavor for future investigations.

With the growing popularity and prevalence of online video gaming, more specifically the engagement with violent video games online, and evidence to suggest that playing against human opponents can heighten the gaming experience, we thought it an important endeavor to investigate whether violent video games played online would exacerbate any negative effects on aggression. As expected, online violent video game play relative to the three remaining experimental increased aggression. However, inasmuch as offline violent video game play relative to the neutral video game conditions also significantly increased aggression, we have to conclude that the violent video game affected aggression but that this effect was not further strengthened by playing the game online. Because this is the first study to have examined the effects of online violent video game play on aggression, we hasten to add that more research is needed before the conclusion is warranted that playing online vs. offline has no consequences on the player's social behavior. For instance, future research may address the effects of online violent video game play on behavioural aggression in the long term. Differences in perceived competition when playing video games online and offline should also be explored. Further to this, future research should investigate the properties of violent video games experienced online that impact on players' aggressive cognitions, affect, physiological arousal and behaviour.

Consideration could also be given to potential positive effects of playing prosocial video games online. Previous research has shown that playing a prosocial video game (where the main objective of the game is to benefit video game characters) increases prosocial behaviour [37]–[39] and empathy [40] and decreases the accessibility of aggressive thoughts [41] and reduces aggressive behaviour [42]. Likewise, playing cooperative team-player (relative to a single-player) video games increases cooperative behaviour and empathy and decreases aggressive cognitions and angry feelings [43]–[49]. It may well be that prosocial and antisocial outcomes are even more affected by prosocial and cooperative video games when played online.

It is important to acknowledge a limitation in regards to the video games selected in this study. The perspective of the FPS is specific, and the authors are unaware of a neutral video game that utilises the first person perspective. It may be possible, in the future, to identify a non-violent first person perspective video game and thus better match the characteristics of the violent and neutral video games. As a result, *LittleBigPlanet 2* was selected for its low PEGI rating and ease of operating the controls (unrelated to game difficulty). It should also be conceded that the two online conditions differed in that participants competed against human opponents in the first-person shooter game, whereas the neutral video game allowed players to play competitively and cooperatively. This possible confound might have led to increased aggression in the online/violent (relative to the offline/violent) video game condition and decreased aggression in the online/neutral (relative to the offline/neutral) video game condition (that is, an interaction between type of video game and setting). However, we did not find this interaction, but simply a main effect of type of video game. In fact, it is compelling that despite these differences in online/offline shooter games that they did not differ in their effect on aggression.

Further to this some concerns have been raised as to the suitability of the chilli sauce paradigm as an accurate measurement of behavioural aggression within the laboratory environment [50]. In addition the current sample size was relatively small and therefore limits the generalisability of the results. Future research should increase the experimental population and may examine the effects of violent video games online on other measures of aggression.

In conclusion this study has identified that increases in aggression are not more pronounced when playing a violent video game online in comparison to playing a neutral video game online. This is an important finding in relation to the growing online community and popularity of violent video games, specifically FPSs, and the potential for subsequent increases in aggression. We think there should be concern about the harmful effects of playing violent video games but it appears that playing the game online does not further exacerbate these effects.

## Supporting Information

File S1 **SPSS data file.**
(SAV)
